# Selective *O*-Alkylation of the Crown Conformer of Tetra(4-hydroxyphenyl)calix[4]resorcinarene to the Corresponding Tetraalkyl Ether

**DOI:** 10.3390/molecules22101660

**Published:** 2017-10-04

**Authors:** Alver Castillo-Aguirre, Zuly Rivera-Monroy, Mauricio Maldonado

**Affiliations:** Departamento de Química, Facultad de Ciencias, Universidad Nacional de Colombia-Sede Bogotá, Carrera 30 No. 45-03, Bogotá 111321, Colombia; aacastilloa@unal.edu.co (A.C.-A.); zjriveram@unal.edu.co (Z.R.-M.)

**Keywords:** resorcinarene, stereoselective, ring-opening reaction, crown conformation

## Abstract

Reactions of glycidyl methacrylate with the crown and chair conformers of tetra(4-hydroxyphenyl)calix[4]resorcinarene were studied. The reactions were done over epoxide groups present in the ester, which can easily undergo an opening reaction with hydroxyl groups in the macrocyclic system. Initially, epoxidation reactions were carried out with pure conformers, and it was observed that the reaction between tetra(4-hydroxyphenyl)calix[4]resorcinarene fixed in the chair conformation does not occur, while for the molecule fixed in the crown conformation only one tetraalkylated derivative was obtained. The obtained product was characterized using IR, ^1^H-NMR, ^13^C-NMR, COSY, HMQC and HMBC techniques. An exhaustive NMR study showed that the reaction is selective at the hydroxyl groups in the lower rim, without affecting the hydroxyl groups in the upper rim. In addition, the RP–HPLC analysis of the epoxidation reaction mixture, using both crown and chair conformers, showed that only the crown conformer reacted under tested conditions. Finally, a comparative study of the reactivity of tetranonylcalix[4]resorcinarene with glycidyl methacrylate showed that the reaction does not take place. Instead, the formation of the tetranonylcalix[4]resorcinarene tetrasodium salt was observed, which confirms that the hydroxyl groups in the upper rim are unreactive under these conditions.

## 1. Introduction

Investigation of resorcinarene derivatives provides a significant contribution to the development of new applications, including separation techniques and heterogeneous catalysts, among others. Resorcinarenes represent a class of macrocyclic phenolic compounds obtained from the condensation reaction of resorcinol with several aromatic and aliphatic aldehydes in acidic solutions [1,2], and they can be modified with various substituents on the upper and lower rim in order to provide specific functionality and selectivity. The many possible structural variations lead to potential applications, such as voltammetric sensors [3], dendrimer synthesis [4,5], dyeing of fibers [6,7], NMR solvating agents [8,9], chemical receptors for molecules and ions [10,11,12], and absorption of heavy metal ions [13,14]. Furthermore, resorcinarene derivatives can be absorbed or covalently bound to multiple surface types. In this way, resorcinarenes are used in HPLC for stationary-phase modification. The process involves the resorcinarene lower rim derivate with polar carbonate groups covalently bonded to a silica substrate [15]. In another example, Tan et al. synthesized and used two new stationary phases, (3-(*C*-methylcalix[4]resorcinarene)-2- hydroxypropoxy)-propylsilyl-appended silica particles (MCR–HPS) and bromoacetate-substituted MCR-HPS particles (BAMCR–HPS), where resorcinarenes are covalently linked by their upper rim to silica particles [16].

Resorcinarene systems present conformations and conformational interconversions, and it has been established that the general stability of the four conformations, in descending order of stability, is cone, partial cone, 1,2-alternate and 1,3-alternate. Of these isomers, the cone conformer (*rccc*) is the most thermodynamically stable compound [17].

Functionalizing of calix[4]resorcinarenes can be done from the starting materials. Varying the nature of the aldehyde substituent group facilitates the modification at the macrocyclic lower-rim system. At the upper rim, the obvious positions for chemical modification are the resorcinol hydroxyl group and the ortho position [18,19,20], the hydroxyl groups of the upper rim being the most common, which can be bridged to form extended cavities or substituted by functional groups for specific applications [21]. An interesting reagent for resorcinarene modification at the upper rim is glycidyl methacrylate (GMA), which can easily undergo a ring-opening reaction with nucleophilic reagents that contain hydroxyl, carboxyl, trysil or amine groups [18,22,23]. The presence of an epoxide ring in GMA favors further chemical modifications for various applications. In recent years, studies have been carried out on the attachment of a hydroxyl group to a heterocyclic ring of GMA under alkaline-catalyzed conditions [24].

In our previous reports about resorcinarene reactivity [19,25], we found that upper-rim modification proceeds efficiently, and they are independent of the resorcinarene starting material conformation. This result is in agreement with reports of other authors [26]. In this paper, we report the reaction of the two conformers of tetra(4-hydroxyphenyl)calix[4]resorcinarene (the chair and crown conformations) with the glycidyl methacrylate epoxide group. Our results showed that this reaction can be strictly dependent on the conformer used for the process. Furthermore, the reaction proceeds selectively at the hydroxyl group in the lower rim, without affecting the hydroxyl groups in the upper rim.

## 2. Results and Discussion

**Scheme 1:**
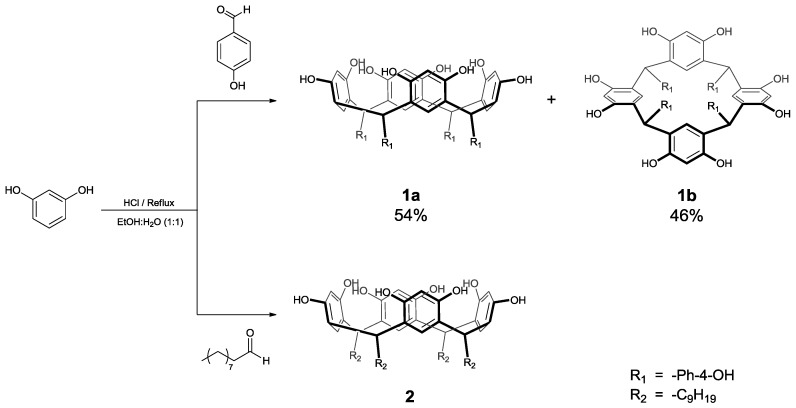
Synthesis of calix[4]resorcinarenes **1a**, **1b** and **2**.

**Figure 1:**
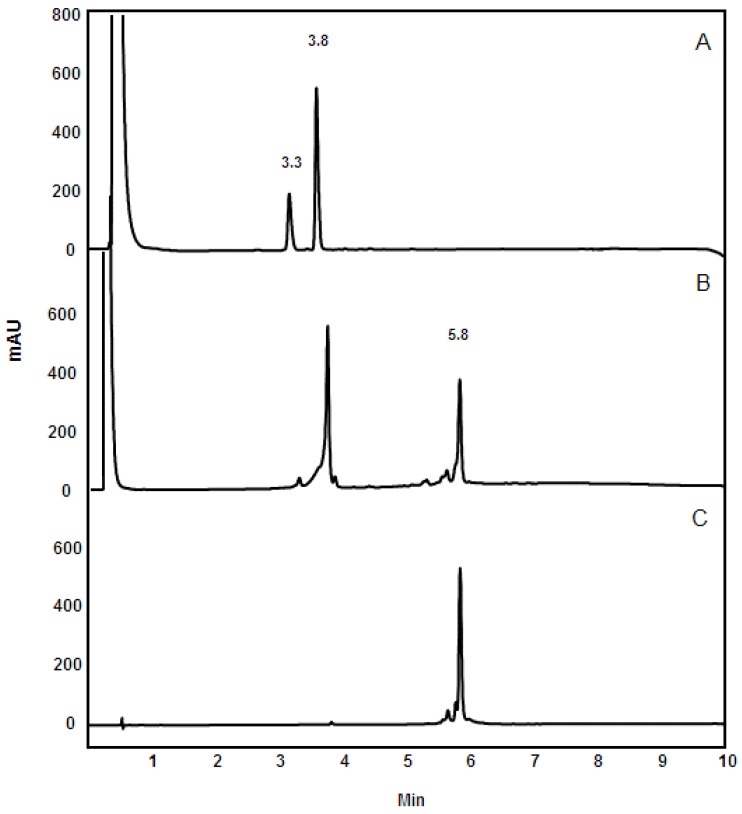
(**A**) Chromatogram of conformational isomers: crown conformation (**1a**), t_R_ = 3.3 min, chair conformation (**1b**), t_R_ = 3.8 min. (**B**) Chromatogram of reaction mixture. (**C**) Chromatogram of pure product **3**, t_R_ = 5.8 min.

Initially, we chose as model substrates the resorcinarenes tetra(4-hydroxyphenyl)calix[4] resorcinarene (*crown*) (**1a**), tetra(4-hydroxyphenyl)calix[4]resorcinarene (*chair*) (**1b**) and tetranonylcalix[4]resorcinarene (**2**), in order to explore the reactivity, selectivity and effects of reaction conditions in the epoxidation using GMA. The synthesis of resorcinarene **2** was done through the acid-catalyzed cyclocondensation of resorcinol with decanaldehyde in a 1:1 mixture of ethyl alcohol and water at 75 °C, in a manner similar to that described in the literature [27]. The product was purified by means of recrystallization. This derivative was characterized using spectral techniques, including FT–IR and ^1^H-NMR (see the Experimental Section). This compound has been previously synthesized by other authors, and our spectroscopic data agree with those reported by them [27,28,29]. The reaction of resorcinol with 4-hydroxybenzaldehyde (Scheme 1) was carried out under the same conditions. RP–HPLC analysis of the reaction mixture showed two products corresponding to the conformational mixture: crown (**1a**) and chair (**1b**) (Figure 1A). These isomers have already been synthesized [30,31,32,33] and possess well-resolved signals in the aromatic region of their ^1^H-NMR spectra, allowing easy assignment of the two conformers and inclusion of their molar ratios in the crude product (1.2/1.0 crown/chair). Finally, the two products (**1a** and **1b**) were separated by means of silica column chromatography using AcOEt:benzene (8:2 *v*/*v*) as mobile phase. The ^1^H-NMR spectrum of the first fraction showed two single peaks at 8.83 and 8.43 ppm, corresponding to two classes of hydroxyl groups, the first signal corresponding to a hydroxyl group in the lower rim and the second signal for the hydroxyl group in the upper rim. Additionally, all the patterns were consistent with the structure of the expected crown conformer **1a**. On the other hand, the spectrum of the second fraction displayed three different hydroxyl moieties at 8.64 ppm, assigned to hydroxyl groups in the lower rim, and two signals at 8.34 and 8.36 ppm, corresponding to two classes of hydroxyl groups attached to resorcinol residues in the macrocyclic system. Careful analysis of all the patterns confirmed the structure of chair conformer **1b**.

**Scheme 2:**
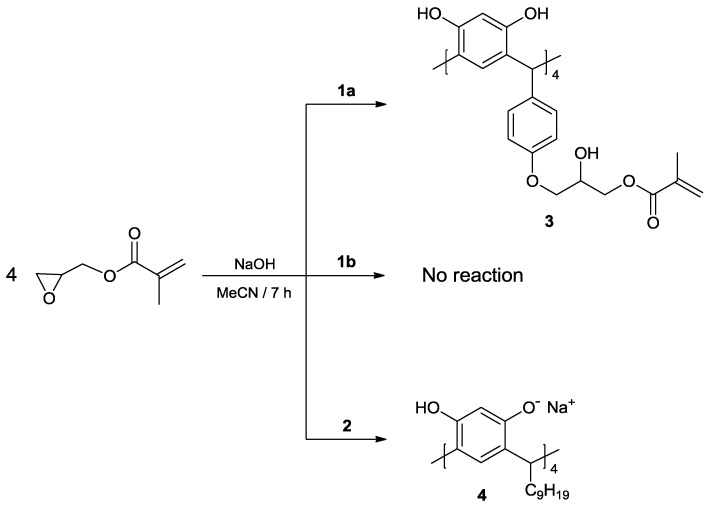
Reaction of glycidyl methacrylate with calix[4]resorcinarenes **1a**, **1b** and **2**.

As mentioned above, the presence of an epoxide ring in GMA favors further chemical modifications for various applications, and the ring-opening reactions of the epoxide require an alkaline medium. In this way, the reactions of resorcinarenes **1a**, **1b** and **2** were done by direct reaction with the glycidyl group through the alkaline-catalyzed ring opening of the epoxy substituent with NaOH, using acetonitrile as a solvent at 57 °C in a manner similar to that described in the literature [24] (Scheme 2). Under these conditions, the reaction of tetra(4-hydroxyphenyl)calix[4]resorcinarene (crown) (**1a**) with a GMA solution in acetonitrile at 80 °C for 7 h afforded a solid product, which was filtered and washed. This solid product was purified by means of recrystallization and was obtained as a pale-orange solid. This derivative was characterized using spectral techniques, including FT–IR, ^1^H-NMR, ^13^C-NMR and 2D-NMR experiments (see the Experimental Section). The FT–IR spectrum of **3** showed carbonyl group (1693 cm^−1^), aromatic ring (1611 cm^−1^), alkyl chain (2929 cm^−1^) and hydroxyl group (3394 cm^−1^) absorptions.

**Figure 2:**
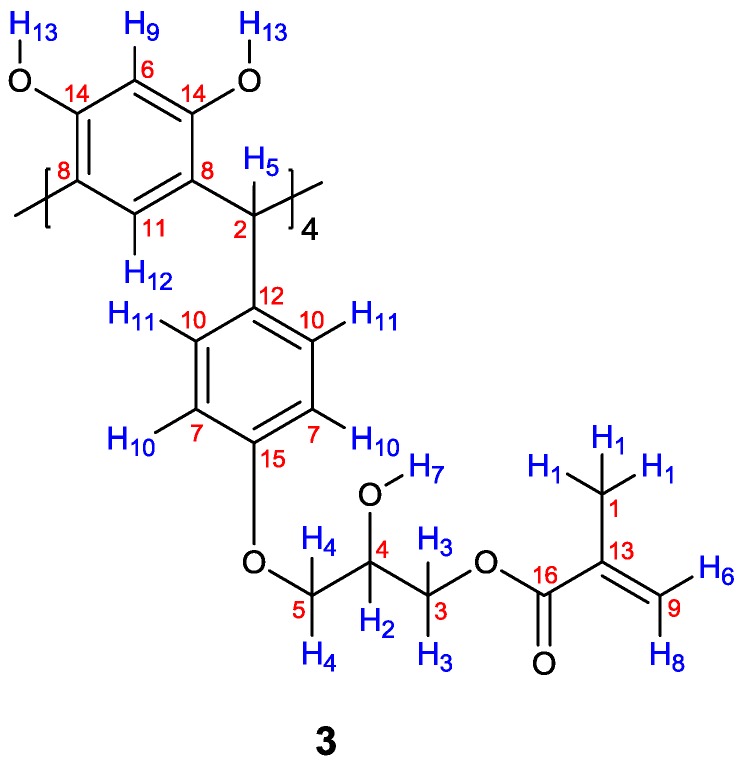
Compound **3** carbon and hydrogen assignments.

In the ^1^H-NMR spectrum of **3**, individual assignments of the protons were made based on their positions, multiplicities, integral values and comparison of spectral data with reported values of similar compounds [18,24,34]. The ^1^H-NMR spectrum of **3** displayed characteristic signals of the glycidyl methacrylate substituent, specifically at 1.91 ppm (methyl groups, H1), 3.88, 4.09 and 4.23 ppm (H2-H4, protons in the open-ring glycidyl fragment) and vinyl protons at 5.69 and 6.11 ppm. In the aromatic region, normally, the ortho and meta protons of the resorcinarene moiety attached to the hydroxyl group give separate signals, as ortho protons are shielded by hydroxyl groups, while meta protons are deshielded and resonate in the upfield region. Given this, the hydrogens of the tetrasubstituted resorcinol units appear at 6.27 (H9) and 6.43 ppm (H12). The signals at 6.31 (H10) and 6.42 (H11) ppm were attributed to the hydrogens in the aromatic ring of the hydroxyphenyl residues. Finally, the signal at 8.40 ppm was assigned to hydroxyl groups in the molecule. In order to confirm the above assignments, the ^1^H–^1^H COSY spectrum was recorded. The correlations for **3** are given in Table 1 and Figure 2; the observed ^1^H–^1^H COSY correlations confirm the assignments made.

The carbon signals in ^13^C-NMR spectrum were unambiguously assigned through 1D- and 2D-NMR experiments, including the HMQC and HMBC spectra. The observed correlations are given in Table 2. The ^13^C-NMR spectrum shows a weak signal at 167.4 ppm with no correlation in the HMQC spectrum. In the HMBC spectrum, this signal shows a correlation with the methyl protons, and hence, this signal must be due to a carbonyl carbon. There are five weak signals at 120.9, 134.4, 135.8, 152.3 and 154.2 ppm. These signals have no correlations in the HMQC spectrum.

In the HMBC spectrum, the signal at 135.8 ppm shows a correlation with the methyl group protons (1.91 ppm). Hence, this signal should be at C-13. The signal at 134.4 ppm has a correlation with H-5, H-10 and H-11 (5.42, 6.31 and 6.42 ppm, respectively). Hence, the signal should be due to the aromatic carbon in the resorcinarene ring (C-12) attached to the bridged methylene. The signal at 152.3 ppm has a correlation with the signals at 5.42 and 6.27 ppm (H-5 and H-9, respectively). Hence, the signals should be due to an aromatic carbon in the resorcinarene ring attached to the hydroxyl group. For the other aromatic residues in the molecule, the HMBC spectrum shows that the signal at 120.9 ppm has correlations with signals at 5.42 and 6.27 ppm (H-5 and H-9, respectively). Therefore, this signal can be assigned to the aromatic carbon attached to the bridged methylene. Finally, the signal at 154.2 ppm shows correlations with the signals at 6.31 and 6.42 ppm (H-10 and H-11, respectively). Therefore, this signal can be assigned to the aromatic carbon substituted by the ether group. The epoxidation of the chair conformer (**1b**) was carried out under the same conditions, but the reaction does not afford the expected tetra-substituted ether; instead, conformer **1b** was quantitatively recovered.

To confirm this behavior, the reaction was carried out with a mixture of the conformers and GMA under the same conditions used with the pure conformers, and the reaction mixture was analyzed by means of RP–HPLC. The chromatographic method was carried out over a monolithic column using a gradient with a mobile phase of acetonitrile and water (containing trifluoroacetic acid 0.05%) applied at a flow rate of 2 mL/min with detection wavelength at 210 nm. Under the mobile phase conditions, elution of the samples was completed in less than 10 min. The chromatogram for the conformational mixture showed that the retention times for **1a** (crown) and **1b** (chair) were 3.3 and 3.8 min, respectively (Figure 1A), although, the conformers have a concentration ratio close to 1:1, and in the chromatographic profile the peaks do not have the same area, indicating that the two conformers do not have the same molar absorptivity coefficient. The chromatographic analysis of the reaction mixture showed the same peaks for conformers **1a** and **1b** (Figure 1B). However, the peak at 3.3 min was noticeably diminished, while the peak at 3.8 min remained unchanged. Additionally, the peak at 5.8 min, corresponding to the retention time of **3**, allowed us to unequivocally establish that *O*-alkylation occurred by direct attack of hydroxyl groups in the lower rim of the crown conformer on the epoxide ring of GMA, and that this process occurred selectively even in the presence of conformer **1b**.

Finally, as expected, when the reaction was performed with **2**, the ring-opening reaction of GMA epoxidation did not proceed, and the only product that could be identified was tetrasodium tetranonylcalix[4]resorcinarene (**4**), which precipitated from the reaction mixture (Scheme 2). The FT–IR spectrum of **4** showed absorptions for C–O stretching (1159 cm^−1^), an aromatic ring (1614 cm^−1^), an alkyl chain (2868, 2930 and 2953 cm^−1^) and a hydroxyl group (3318 cm^−1^). The ^1^H-NMR spectrum displayed characteristic signals of nonyl chains (0.85, 1.21, 2.00 ppm), a methylene bridge fragment between the aromatic rings (4.14 ppm), and the aromatic hydrogens of the tetrasubstituted resorcinol unit (6.06 and 7.02 ppm). For hydroxyl protons, the signal at 9.67 ppm integrates for four protons. The other protons were replaced by sodium ions. The ^13^C-NMR spectrum in DMSO-*d*_6_ showed fourteen signals, which agreed with the structure of compound **4**, that is, it displayed nine signals for nonyl chains as shown in the experimental section. In this way, the signal at 34.0 ppm confirmed the presence of the methylene bridge fragment between the aromatic rings, and the aromatic carbons appeared at 103.6, 123.8 and 152.9 ppm. RP–HPLC analysis of the reaction mixture allowed us to establish that GMA did not react, as it was recovered quantitatively.

Although the epoxide group present in GMA can easily undergo an opening reaction with nucleophilic reagents that contain hydroxyl groups, such as resorcinarenes, NMR analysis of **3** and **4** showed that the reactions do not proceed at the hydroxyl groups in the upper rim of resorcinarenes **1a** or **2**, and the reaction with **1b** does not proceed. When the ring-opening reaction of GMA was conducted in the presence of **1a**, 90% of the GMA was consumed after 7 h, and a product of *O*-alkylation of the hydroxyl groups in the lower rim was obtained, with 22% yield. The results observed between GMA and the studied resorcinarenes make it possible to establish that the reaction, under the described conditions, is regioselective and stereoselective, exhibiting a great dependence on the starting conformation of the resorcinarene. Firstly, the observed regioselectivity with conformer **1a** can be explained by the different reactivity of hydroxyl groups. In **1a**, two classes of hydroxyl groups are observed: eight hydroxyl groups from resorcinol residues in the upper rim of the resorcinarene system, which are stabilized by OH···O hydrogen bonds, forming a strong interaction and making them less reactive than the four hydroxyl groups from the pendant substituent (lower rim) under the reaction conditions. This tendency is confirmed by the results observed in the reaction of **2** with glycidyl methacrylate, which also does not generate the product of *O*-alkylation of hydroxyl groups and which supports our finding that the regioselectivity in the crown conformer of tetra(4-hydroxyphenyl)calix[4]resorcinarene is due to the strong hydrogen bonding in the upper rim between hydroxyl groups [2,27,35,36].

Secondly, the reaction is stereoselective. The results of the epoxidation of tetra(4-hydroxyphenyl)calix[4]resorcinarene immobilized in the crown (**1a**) and in the chair (**1b**) conformations revealed a different reactivity, as has been observed in other similar cases [37,38,39]. While **1a** produces the ether derivative **3**, the epoxidation of **1b** does not proceed, and this tendency is confirmed by the results observed in the reaction of glycidyl methacrylate with the conformational mixture. A possible explanation of this behavior can be proposed: the strong interaction between the lower- and upper-rim hydroxyl groups in the chair conformation of **1b** [35], which also did not generate the product of *O*-alkylation of hydroxyl groups, because the conformation was maintained in a circular hydrogen array [40,41].

## 3. Materials and Methods 

### 3.1. General Experimental Information

IR spectra were recorded on a Thermo Fisher Scientific Nicolet iS10 FT–IR spectrometer with a Monolithic Diamond ATR accessory and absorption in cm^−1^. ^1^H- and ^13^C-NMR spectra were recorded at 400 MHz on a Bruker Avance 400 instrument. Molar mass was determined on a MALDI–TOF spectrometer (Bruker Daltonics, Bremen, Germany) using 4-nitroaniline as a matrix for the desorption/ionization process. RP–HPLC analyses were performed over a Chomolith^®^ C18 column (Merck, Kenilworth, NJ, USA, 50 mm) using an Agilent 1200 Liquid Chromatograph (Agilent, Omaha, NE, USA). Chemical shifts are reported in ppm, using the solvent residual signal. The elemental analysis for carbon and hydrogen was carried out using a Thermo Flash 2000 Elemental Analyzer. The synthesis of these macrocycles was performed by means of the reaction between resorcinol and different aldehydes under acidic conditions [42,43]. Used aldehydes were n-decanaldehyde and 4-hydroxybenzaldehyde.

### 3.2. Synthesis of Calix[4]resorcinarenes

A resorcinol solution (10 mmol) and aldehyde (10 mmol) in ethanol:water (1:1) (20 mL) was added drop by drop to hydrochloric acid (2 mL) and was reacted at reflux with constant stirring for 1–6 h. The reaction mixture was cooled in an ice bath, and the solid material that was formed was filtered and washed with water to remove traces of acid. The filtrate was dried under vacuum and was characterized by means of IR and ^1^H-NMR.

*Tetra(4-hydroxyphenyl)calix[4]resorcinarene* (*crown*) (**1a**) was obtained as pink solid at a yield of 54% (determined by NMR). Mp > 250 °C decomposition. IR (KBr/cm^−1^): 3384 (O-H), 1249 (C-O); ^1^H-NMR, DMSO-*d*_6_, δ (ppm): 5.53 (s, 4H, ArCH), 6.08 (s, 4H, ArH, ortho to OH), 6.47 (d, 8H, *J* = 8 Hz, ArH), 6.50 (s, 4H, ArH, meta to OH), 6.63 (d, 8H, *J* = 8 Hz, ArH), 8.43 (s, 8OH, ArOH), 8.83 (4OH, ArOH); ^13^C-NMR, δ (ppm): 40.6 (ArCH), 102.9 (resorcinol C-2), 114.0 (resorcinol C-5), 121.0 (hydroxyphenyl C-3), 129.6 (hydroxyphenyl C-2), 136.0 (hydroxyphenyl C-4), 152.2 (resorcinol C-4), 152.3 (hydroxyphenyl C-1), 154.5 (resorcinol C-1).

*Tetra(4-hydroxyphenyl)calix[4]resorcinarene (chair)* (**1b**) was obtained as a pink clear solid at a yield of 46% (determined by NMR). Mp > 250 °C decomposition. IR (KBr/cm^−1^): 3384 (O-H), 1249 (C-O); ^1^H-NMR, DMSO-*d*_6_, δ (ppm): 5.42 (s, 4H, ArCH), 5.92 (s, 2H, ArH, ortho to OH), 6.09 (s, 2H, ArH, ortho to OH), 6.27 (s, 2H, ArH, meta to OH), 6.30 (s, 2H, ArH, meta to OH), 6.31 (d, 8 H, *J* = 8 Hz, ArH), 6,42(d, 8H, *J* = 8 Hz, ArH), 8.32 (s, 4OH, ArOH), 8.34 (s, 4OH, ArOH), 8.64 (s, 4 OH, ArOH); ^13^C-NMR, δ (ppm): 41.2 (ArCH), 113.9 (resorcinol C-2), 120.8 (hydroxyphenyl C-3), 121.9 (hydroxyphenyl C-2), 129.9 (resorcinol C-5), 134.6 (hydroxyphenyl C-4), 152.4 (resorcinol C-4), 152.7 (hydroxyphenyl C-1), 154.4 (resorcinol C-1).

*Tetranonylcalix[4]resorcinarene* (**2**) was obtained as a yellow solid at a yield of 88%. Mp > 250 °C decomposition. IR (KBr/cm^−1^): 3258 (O-H), 1171 (C-O); ^1^H-NMR, CDCl_3_, δ (ppm): 0.88 (t, 12H, *J* = 8 Hz, CH_3_), 1.27 (m, 64H, (CH_2_)_8_), 4.30 (t, 4H, CH), 6.11 (s, 4H ortho to OH), 7.21 (s, 4H meta to OH), 9.32 (s, 4H, OH), 9.60 (s, 4H, OH); ^13^C-NMR, δ (ppm): 14.3 (CH_3_), 22.9 (CH_2_), 28.2 (CH_2_), 29.5 (CH_2_), 29.7 (CH_2_), 29.8 (CH_2_), 29.9 (CH_2_), 30.0 (CH_2_), 32.1 (CH_2_), 33.4 (CH), 124.0, 125.0, 150.5 and 150.8 (resorcinol ring).

### 3.3. Reaction of calix[4]resorcinarenes **1a**, **1b** and **2** with GMA 

Reactions were carried out following the method outlined in the literature [24]. In brief, calix[4]resorcinarene (1 mmol) was reacted with glycidyl methacrylate (GMA) (8–12 mmol) under catalysis of solid NaOH (4 mmol) in MeCN (45 mL) in a 250 mL two-neck round-bottom flask at 80 °C for 7 h with constant stirring and reflux under a nitrogen atmosphere. The reaction mixture was cooled in an ice bath, and the solid material formed was filtered and washed with MeCN to remove traces of NaOH. The filtrate was dried under vacuum and was characterized by means of IR, ^1^H-NMR, ^13^C-NMR, COSY, HMQC and HMBC.

*2,8,14,20-Tetra(4-{2-hydroxy-3-[(2-methylacriloyl)oxy]propoxy}phenyl)calix[4]resorcinarene* (**3**) was obtained as a pale orange powder at a yield of 22%. Mp > 250 °C decomposition. IR (KBr/cm^−1^): 3394 (O-H), 1693 (C=O), 1238 (C-O); ^1^H-NMR, DMSO-*d*_6_, δ (ppm): 1.91 (12H, CH_3_), 3.88 (4H, OCH), 4.09 (8H, COCH_2_), 4.23 (8H, ArOCH_2_), 5.42 (4H, ArCH), 5.69 (4H, C_sp2_H), 5.91 (4H, COH), 6.11 (4H, C_sp2_H), 6.27 (4H, resorcinol HC-2), 6.31 (8H, *J* = 8 Hz, oxyphenyl HC-2), 6.42 (8H, *J* = 8 Hz, oxyphenyl HC-3), 6.43 (4H, resorcinol HC-5), 8.40 (8H, resorcinol ArOH); ^13^C-NMR, δ (ppm): 18.0 (CH_3_), 41.2 (ArCH), 66.3 (COCH_2_), 68.8 (COH), 71.4 (ArOCH_2_), 101.6 (resorcinol C-2), 113.8 (oxyphenyl C-2), 120.9, (resorcinol C-4), 125.9 (H_2_C_sp2_), 129.7 (oxyphenyl C-3), 129.8 (resorcinol C-5), 134.4 (oxyphenyl C-4), 135.8 (C_sp2_), 152.3 (resorcinol C-1), 154.2 (oxyphenyl C-1), 167.4 (C=O). MALDI–TOF MS (4-nitroaniline) analysis shows a signal at m/z = 1448.57 corresponding to [M + Na]^+^ (calc. mass for M (C_80_H_80_O_24_): 1425.47). Anal. calcd. for (molecular formula, C_80_H_80_O_24_): C = 67.41, H = 5.66; found: C = 66.96, and H = 5.73.

*Tetrasodium 2,8,14,20-tetranonylpentacyclo[19.3.1.1^3,7^.1^9,13^.1^15,19^]octacosa-1(25),3,5,7(28),9,11,13(27), 15,17,19(26),21,23-dodecaeno-4,10,16,22-tetraol-6,12,18,24-tetrakis(olate)* (**4**) was obtained as a cream-colored powder at a yield of 42%. Mp < 250°C decomposition. IR (KBr/cm^−1^): 3319 (O-H), 1210 (C-O); ^1^H-NMR, DMSO-*d*_6_, δ (ppm): 0.85 (t, 12H, CH_3_), 1.21 (m, 56H, CH_2_), 2.00 (q, 8H, CH_2_), 4.14 (t, 4H, CH), 6.06 (s, 4H, ortho to OH), 7.02 (s, 4H, meta to OH), 9.57 (s, 4H, OH); ^13^C-NMR, δ (ppm): 14.4 (CH_3_), 19.2 (CH_2_), 22.7 (CH_2_), 28.5 (CH_2_), 29.4 (CH_2_), 29.8 (CH_2_), 30.0 (CH_2_), 32.0 (CH_2_), 33.9 (CH_2_), 34.0 (CH), 103.6, 123.8 and 152.9 (resorcinol ring). Anal. calcd. for (molecular formula, C_64_H_92_O_8_Na_4_): C = 71.08, H = 8.58; found: C = 70.92, and H = 8.62.

### 3.4. Reversed-Phase HPLC Analysis

The RP–HPLC analysis was performed on a Merck Chromolith^®^ C18 (50 × 4.6 mm) column using an Agilent 1200 liquid chromatograph (Agilent, Omaha, NE, USA) with UV-vis detector (210 nm). For the analysis of products (10 μL, 1 mg/mL), a linear gradient was applied from 5% to 70% Solvent B (0.05% TFA in MeCN) in Solvent A (0.05% TFA in water) in 11.5 min at a flow rate of 2.0 mL/min at room temperature.

## 4. Conclusions 

Epoxidation reactions of conformers of tetra(4-hydroxyphenyl)calix[4]resorcinarene (crown and chair) were carried out using GMA as the epoxidating agent under alkaline-catalyzed conditions. The upper rim of unsubstituted tetra(4-hydroxyphenyl)calix[4]resorcinarene immobilized in the crown conformation could be regioselectivity alkylated to give a tetraalkyl ether, which was analyzed by means of ^1^H-NMR, ^13^C-NMR and 2D-NMR. Although the formation of polyalkylated products is theoretically possible, it was found that the corresponding alkylated ether is not formed with the chair conformer. RP–HPLC analysis of the epoxidation reaction with a conformational mixture (crown and chair) confirmed this behavior under the same reaction conditions. Finally, a comparative study of the reactivity of tetranonylcalix[4]resorcinarene with glycidyl methacrylate confirmed that the hydroxyl groups in the upper rim are unreactive under these conditions. The results observed between the GMA and the studied resorcinarenes make it possible to establish that under the described conditions the reaction is regioselective and stereoselective, exhibiting a great dependence on the starting conformation of the resorcinarene.

## Figures and Tables

**Table 1:** Principal correlations for compound **3** in the COSY spectrum.

Proton	δ (ppm)	Correlation
1	1.91	6 (5.69)
1	1.91	8 (6.11)
2	3.88	3 (4.09)
2	3.88	4 (4.23)
6	5.69	8 (6.11)
10	6.31	11 (6.42)

**Table 2:** Correlations for compound **3** in the HMQC and HMBC spectra.

Carbon	δ (ppm)	Correlation HMQC	Correlation HMBC
1	18.0	1 (1.91)	6 (5.69), 8 (6.11)
2	41.2	5 (5.42)	11 (6.42), 12 (6.43)
3	66.3	3 (4.09)	2 (3.88)
4	68.8	2 (3.88)	4 (4.23)
5	71.4	4 (4.23)	3 (4.09)
6	101.6	9 (6.27)	13 (8.40)
7	113.8	10 (6.31)	11 (6.42)
8	120.9	-	5 (5.42), 9 (6.27), 13 (8.40)
9	125.9	6 (5.69), 8 (6.11)	1 (1.91)
10	129.7	11 (6.42)	5 (5.42), 10 (6.31)
11	129.8	12 (6.43)	5 (5.42)
12	134.4	-	5 (5.42), 10 (6.31), 11 (6.42)
13	135.8	-	1 (1.91)
14	152.3	-	5 (5.42), 9 (6.27), 13 (8.40)
15	154.2	-	10 (6.31), 11 (6.42)
16	167.4	-	1 (1.91), 3 (4.09), 6 (5.69), 8 (6.11)

## Supplementary Materials

The Supplementary Materials are available online.
