# Supplementary material for: Selective O-Alkylation of the Crown Conformer of Tetra(4-hydroxyphenyl)calix[4]resorcinarene to the Corresponding Tetraalkyl Ether
Source: Molecules. 2017 Oct 4;22(10):1660. doi: 10.3390/molecules22101660 (PMC6151838; doi:10.3390/molecules22101660)
Supplement: Supplementary file 1 [file molecules-22-01660-s001.pdf]

Supplementary Materials: Selective epoxidation of tetra(4-hydroxyphenyl)calix[4]resorcinarene (*crown*) to corresponding tetraalkyl ether

Alver Castillo-Aguirre, Zuly Rivera-Monroy, Mauricio Maldonado

Table of Contents:

Figure S1. Structure of compound **3**

Figure S2. UV spectrum of compound **3**

Figure S3. IR spectrum of compound **3**

Figure S4. ^{1}H-NMR spectrum (400MHz, DMSO-d_6, 303 K) of compound **3**

Figure S5. ^{13}C-NMR, DEPT 135, DEPT 90 and DEPT 45 spectrum (400MHz, DMSO-d_6, 303 K) of compound **3**

Figure S6. ^{1}H-^{1}H COSY NMR spectrum (400MHz, DMSO-d_6, 303 K) of compound **3**

Figure S7. HMQC NMR spectrum (400MHz, DMSO-d_6, 303 K) of compound **3**

Figure S8. HMBC NMR spectrum (400MHz, DMSO-d_6, 303 K) of compound **3**

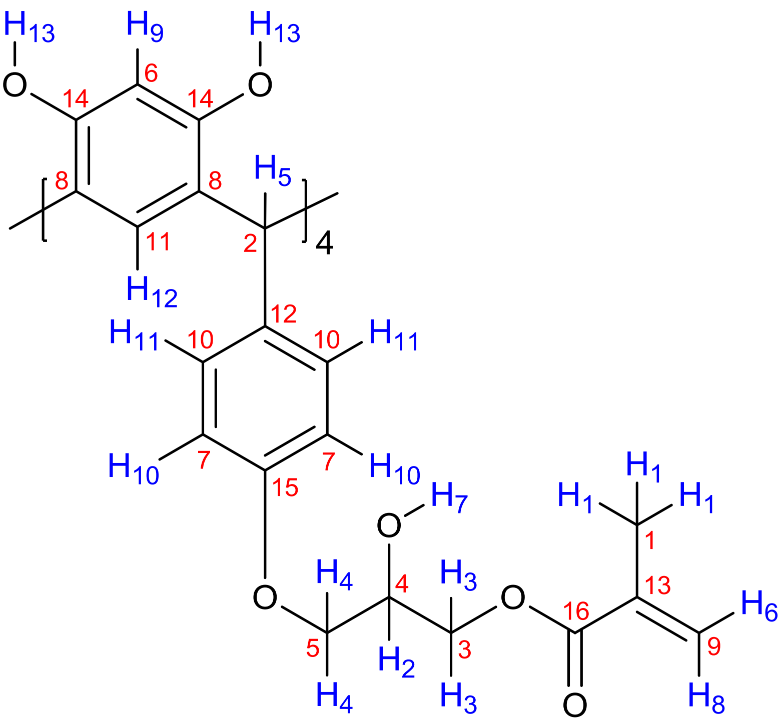

Figure S1. Structure of compound **3.**

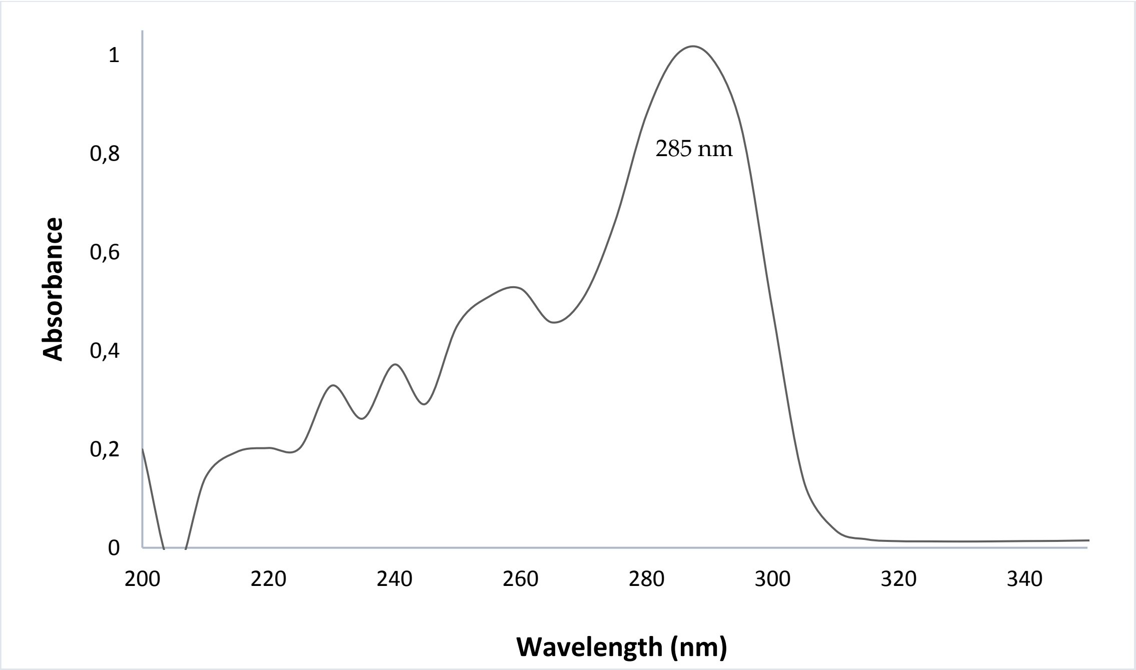

Figure S2. UV spectrum of compound **3**.

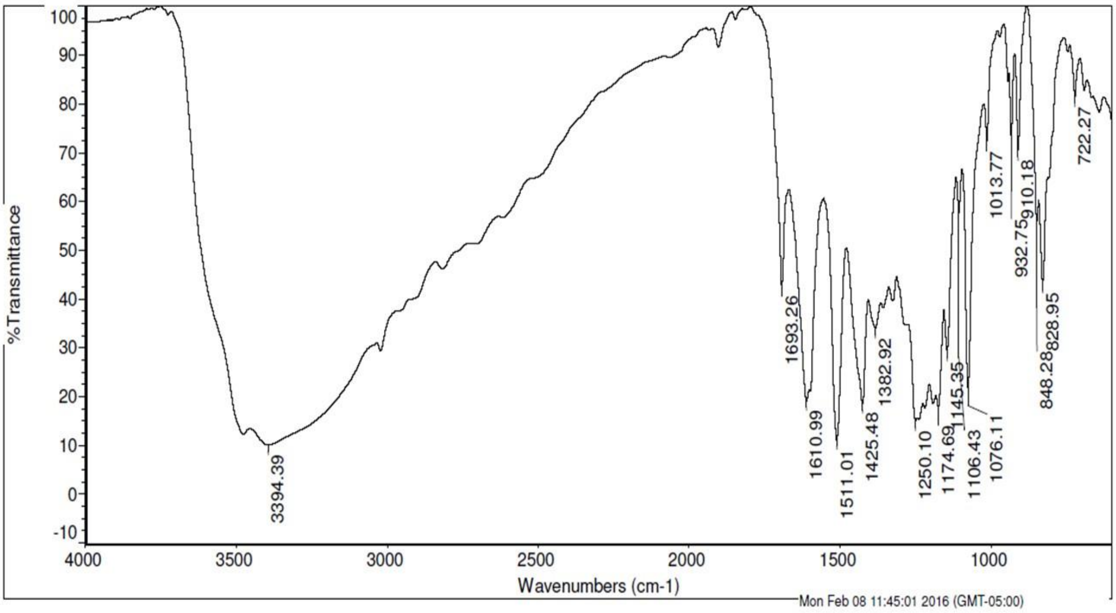

Figure S3. IR spectrum of compound **3**.

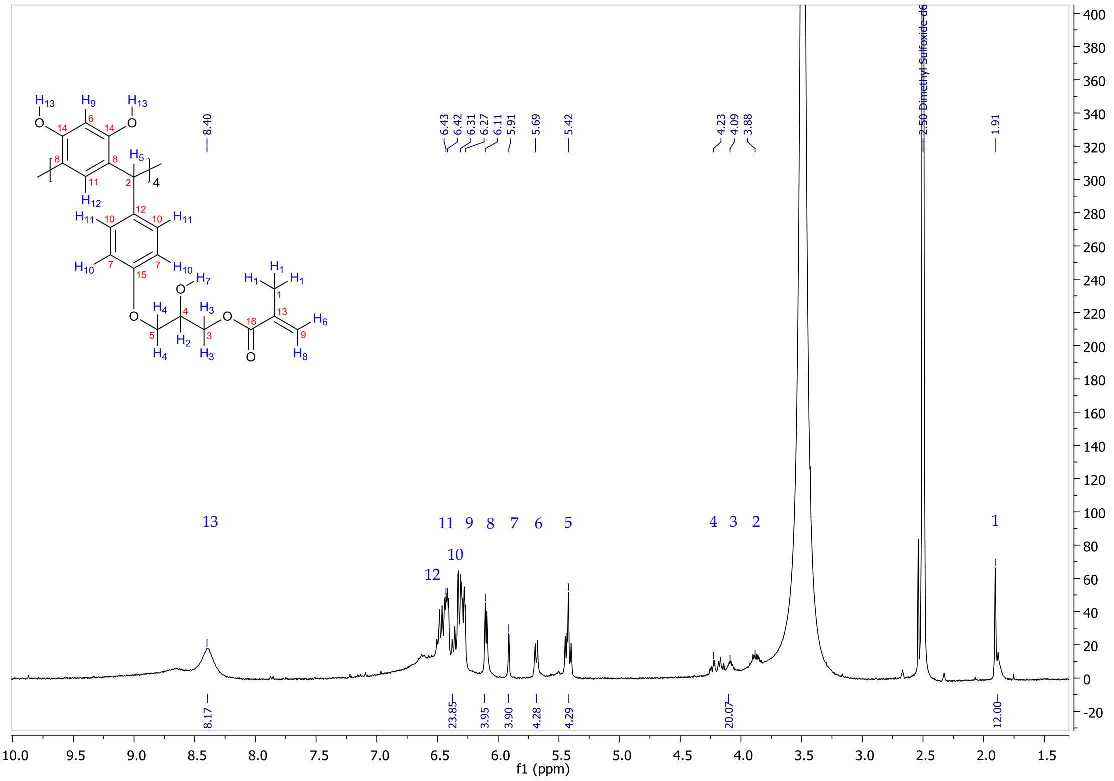

Figure S4. ^{1}H-NMR spectrum (400MHz, DMSO-d_6, 303 K) of compound **3**.

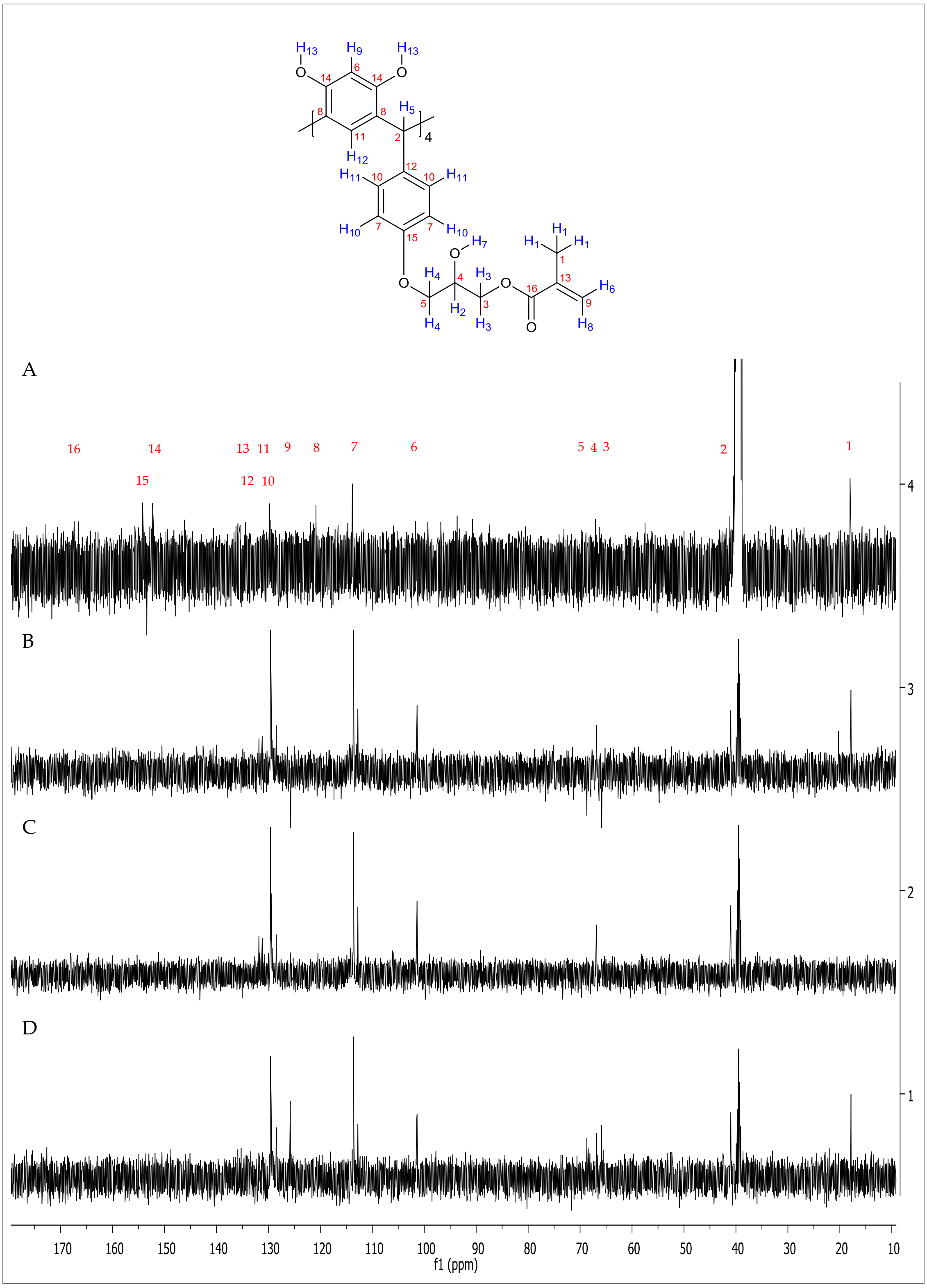

Figure S5. (A) ^{13}C-NMR, (B) DEPT 135, (C) DEPT 90 and (D) DEPT 45 spectrum (400MHz, DMSO-d_6, 303 K) of compound **3**.

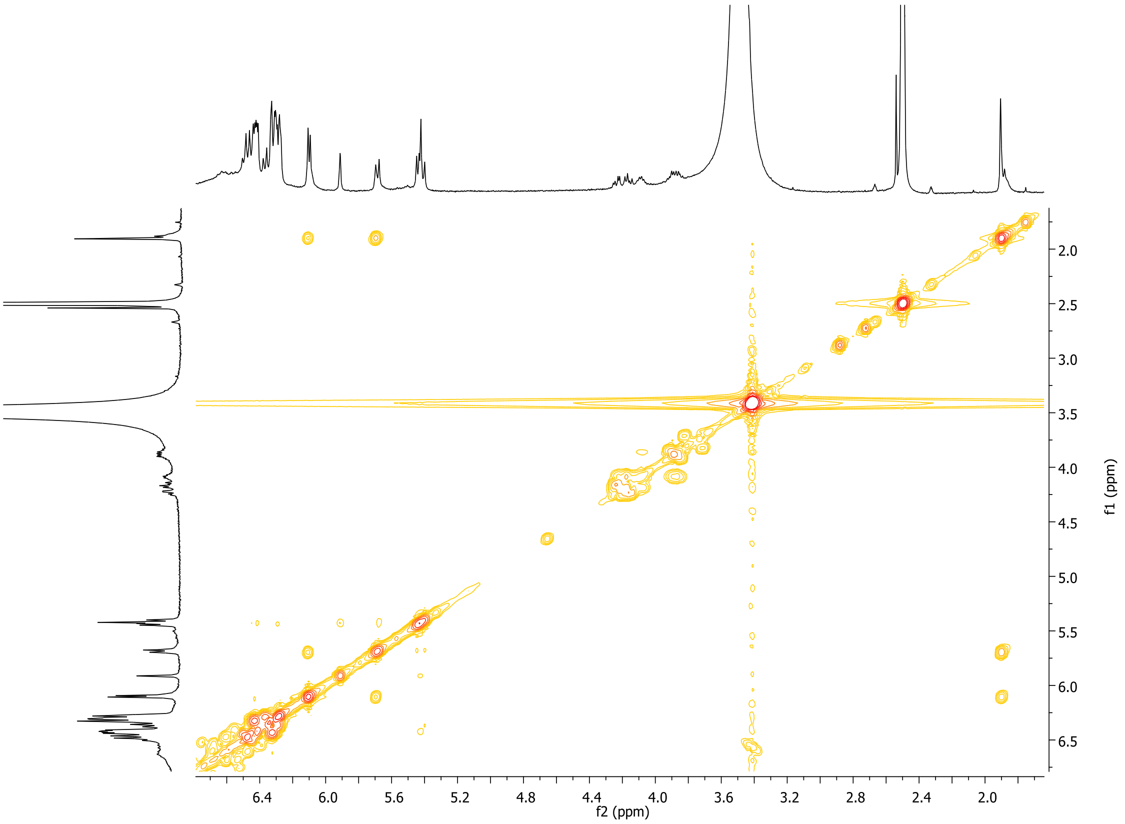

Figure S6. ^1H-^1H COSY NMR spectrum (400MHz, DMSO-*d*6, 303 K) of compound **3**.

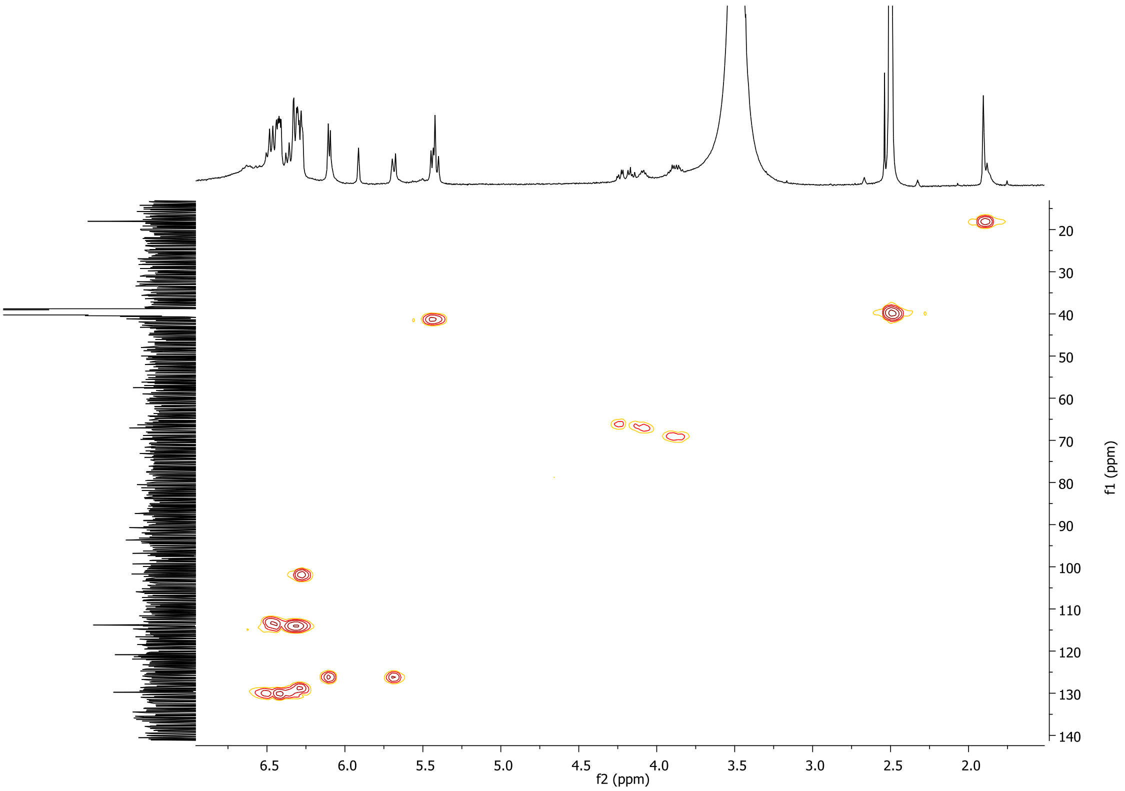

Figure S7. HMQC NMR spectrum (400MHz, DMSO-*d*6, 303 K) of compound **3**

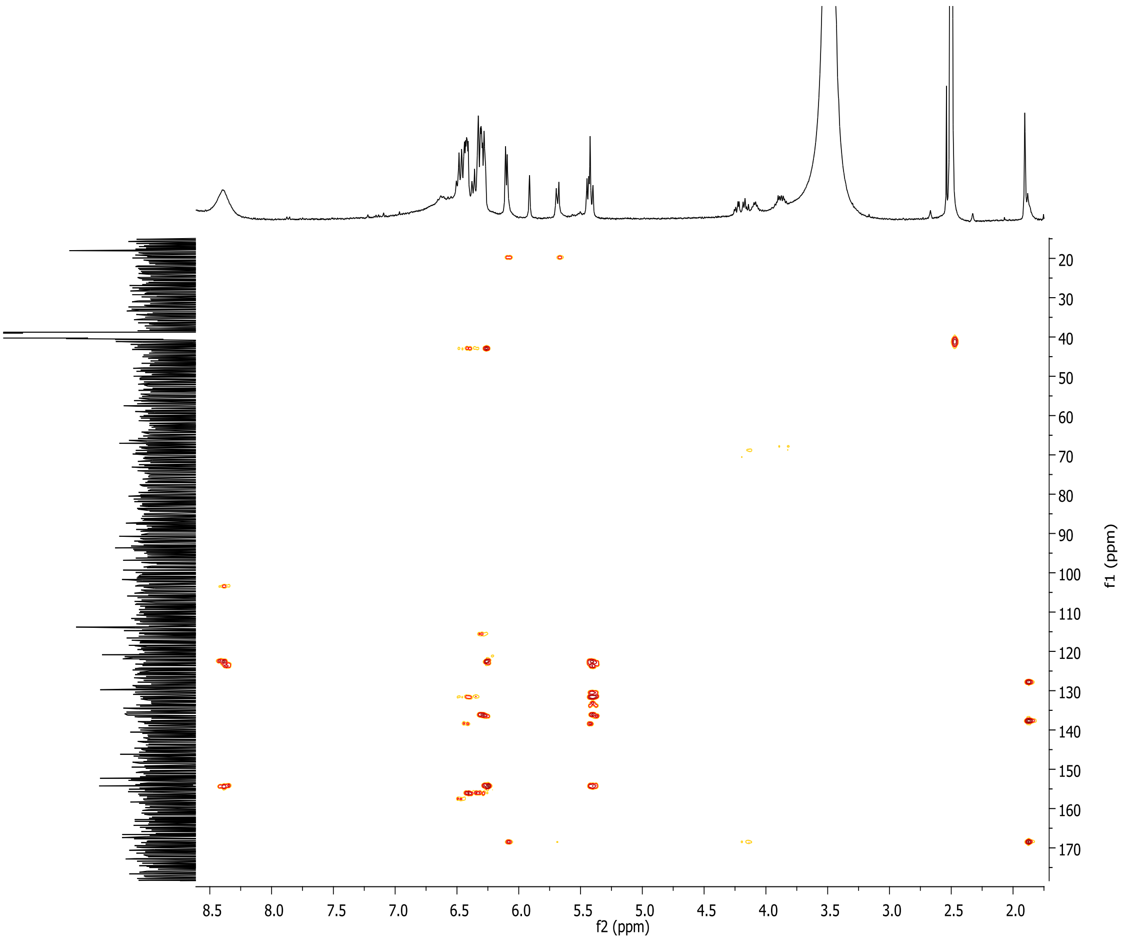

Figure S8. HMBC NMR spectrum (400MHz, DMSO-d_6, 303 K) of compound **3**
